# Deletion of mitochondrial uncoupling protein 2 exacerbates mitophagy and cell apoptosis after cerebral ischemia and reperfusion injury in mice

**DOI:** 10.7150/ijms.49849

**Published:** 2020-10-16

**Authors:** Maotao He, Ting Zhang, Yucheng Fan, Yanmei Ma, Jianzhong Zhang, Li Jing, P. Andy Li

**Affiliations:** 1 Department of Pathology, General Hospital of Ningxia Medical University, Yinchuan, Ningxia 750004, China.; 2 School of Basic Medical Sciences, Department of Pathology, Ningxia Medical University; Ningxia Key Laboratory of Vascular Injury and Repair, Yinchuan, Ningxia 750004, China.; 3 Department of Pharmaceutical Sciences, Biomanufacturing Research Institute and Technological Enterprise (BRITE), College of Health and Sciences, North Carolina Central University, Durham, NC 27707, USA.

**Keywords:** uncoupling protein 2, cerebral ischemia/reperfusion, mitophagy, autophagy, apoptosis, ROS

## Abstract

**Objective:** Uncoupling protein 2 (UCP2) is a member of inner mitochondrial membrane proteins and deletion of UCP2 exacerbates brain damage after cerebral ischemia/reperfusion (I/R). Nevertheless, its functional role during cerebral I/R is not entirely understood. The objective of present study was to explore the influence of UCP2 deletion on mitochondrial autophagy (mitophagy) and mitochondria-mediated cell death pathway after cerebral I/R.

**Methods:** UCP2^-/-^ and wildtype (WT) mice were subjected to 60 min middle cerebral artery occlusion (MCAO) and allowed reperfusion for 24 hours. Infarct volume and histological outcomes were assessed, reactive oxygen species (ROS) and autophagy markers were measured, and mitochondrial ultrastructure was examined.

**Results:** Deletion of UCP2 enlarged infarct volume, increased numbers of necrotic and TUNEL positive cells, and significantly increased pro-apoptotic protein levels in UCP2^-/-^ mice compared with WT mice subjected to the same duration of I/R. Further, deletion of UCP2 increased ROS production, elevated LC3, Beclin1 and PINK1, while it suppressed p62 compared with respective WT ischemic controls. Electron microscopic study demonstrated the number of autophagosomes was higher in the UCP2^-/-^ group, compared with the WT group.

**Conclusions:** It is concluded that deletion of UCP2 exacerbates cerebral I/R injury via reinforcing mitophagy and cellular apoptosis in mice.

## Introduction

Uncoupling protein 2 (UCP2) is a member of inner mitochondrial membrane proteins. It stabilizes the inner mitochondrial membrane potential and reduces the reactive oxygen species (ROS) generation 1, 2. Several studies have shown that UCP2 plays important roles in regulating oxidative stress and mitochondrial apoptosis signaling. Abundant evidence also demonstrated that UCP2 plays a significant role in the pathogenesis of cerebral ischemia/reperfusion (I/R) damage 3, 4, 5. Our previous studies have shown that deletion of UCP2 gene increases infarct volume after ischemic stroke; and that overexpression of UCP2 reduces ischemic infarct volume 6 and activates cell survival factors 7. Moreover, deletion of UCP2 has been reported to be associated with mitochondrial damage in cerebral I/R injury 8.

Autophagy is a degradation process of lysosomal metabolism, which is responsible for the degradation of aged or deteriorated organelles and cytoplasmic compositions via lysosomal engulfment into autophagosomes. Mitochondrial autophagy (aka mitophagy) is one of the most important patterns of autophagy. It refers to selective sequestration of mitochondria by autophagosomes, which subsequently were delivered to lysosomes for destruction 9. The roles of mitophagy in the cerebral I/R have been intensively studied. Recent reports have shown that I/R-induced mitophagy 10. Nonetheless, the role of mitophagy in cerebral I/R is still controversial. While there were studies showed that activation of mitophagy attenuated mitochondrial dysfunction after cerebral I/R 11, 12, 13, others suggested that enhanced mitophagy led to cell death and attenuation of mitophagy protected neurons from cerebral injury 14, 15. Therefore, overactivation of mitophagy may degrade essential mitochondria and causes cell death 16. Recent studies have also shown that mitophagy interacts with ROS and apoptosis in stroke model, especially in PINK1-Parkin-dependent mitochondria 13, 17. Our previous studies have revealed that UCP2 attenuates mitochondrial dysfunction and reduces ROS generation from cerebral damage induced by cerebral I/R 6. However, it is not clear that whether UCP2 could regulate mitophagy in the setting of cerebral I/R injury. Therefore, the objective of this study was to explore the impact of UCP2 deletion on mitophagy and cell death after cerebral I/R.

## Methods

### Materials

2,3,5-Triphenyl Tetrazolium Chloride (TTC) was obtained from Sigma. TUNEL Assay Kit was purchased from Roche (Mannheim, Germany). A Reactive Oxygen Species Assay Kit was obtained from Beyotime (Jiangsu, China). Antibodies against Caspase 3, cleaved Caspase 3, apoptosis protease-activating factor-1 (Apaf-1), and cytochrome c (Cyto c) were purchased from Cell Signaling Technology (Danvers, MA). LC3B (ab51520) and p62 (ab109012) antibodies were purchased from Abcam. Beclin1 (11306-1-AP), PINK1 (23274-1-AP), and MnSOD (24127-1-AP) antibodies were purchased from Proteintech. Anti-β-actin antibody was purchased from Bios (Beijing, China).

### Animals and groups

Male UCP2^-/-^ (n=24) and wildtype C57BL/6 mice (WT, n=48) at age of 6-8 weeks were used in this study. All animal procedures were performed in accordance with the NIH Guide for Care and Use of Laboratory Animals and were approved by the Institutional Animal Care and Use Committee at Ningxia Medical University. Breeding pairs of UCP2^-/-^ mice were obtained from the Jackson laboratory and their off-springs were genotyped. The mice were maintained in a specific pathogen-free environment in Laboratory Animal Center of Ningxia Medical University (Yinchuan, China) with controlled temperature, humidity, and 12:12 hour light and dark cycle. Animals were divided to three groups: Sham group (n=24), MCAO WT group (n=24) and MCAO UCP2^-/-^ group (n=24): UCP2^-/-^ mice being subjected to 60 min MCAO plus 24 h reperfusion.

### Ischemic model

Cerebral ischemia was induced by occlusion of right middle cerebral artery using a nylon filament. Briefly, animals were anesthetized with isoflurane for induction and maintained at 1.0 - 1.5% during the surgical procedures. The internal carotid artery (ICA), external carotid artery (ECA), and the common carotid artery (CCA) were isolated through a ventral cervical midline incision, then the right CCA was ligated. A small incision was made on the CCA and a filament (Doccol corporation, USA), which had a distal cylinder of silicon rubber with a diameter of 0.21±0.02 mm. After 60 min occlusion, the filament was withdrawn to achieve recirculation. During the period of surgery, the body temperature of the mice was maintained at approximately 37±0.5°C with a heating pad and lamp and was monitored by a rectal temperature probe. The mice were subjected to a neurological examination after 24 h reperfusion using Zea-Longa score, where 0, no neurological deficits; 1, failure to fully extend left forepaw; 2, circling to the left; 3, falling to the left; 4, unable to walk spontaneously and exhibiting depressed levels of consciousness.

### Measurement of infarct volume and edema

The mice were sacrificed at 24 h after MCAO and whole brains were dissected coronally into 1-mm brain slices using a stainless brain matrix (68707, RWD, Shenzhen). The brain slices were immediately incubated with 2% 2, 3, 5-triphenyltetrazolium chloride (TTC) at 37 °C for 15 min and then fixed in 4% paraformaldehyde. The pale color areas were defined as infarct area. Areas of infarct tissue were measured using NIH Image J software (rsb.info.nih.gov/nih-image) and infarct volumes were calculated from all sections with corrections of intersectional distance. The infarct volume was expressed as percentage of infarct tissue relative to total brain tissue. Brain edema was assessed after 24 h reperfusion. The relative edema volume (%) was calculated as: (ipsilateral hemisphere volume - contralateral hemisphere volume)/contralateral hemisphere volume ×100%.

### ROS detection

The mice brain sections were cultured and incubated with 10 μM/L of dihydroethidium (DHE) for 30 min at 37 °C. Red precipitations that reflect intracellular superoxide production were captured and measured with an Olympus FluoView1000 Laser Scanning Confocal Microscope (using ex/em λ=480 nm/535 nm for DHE) and presented as relative fluorescent intensity.

### TUNEL staining

Terminal deoxynucleotidyl transferase mediated dUTP nick‑end labeling (TUNEL) staining was used to detect apoptosis (Roche, #11684795910) according to the manufacturer's protocol. The number of TUNEL-positive cells was counted in five microscopic fields at 400X.

### Immunofluorescence staining

The brain sections were submerged in citrate buffer for antigen retrieval. The protein levels of LC3B (1:100, ab51520, Abcam), Beclin1 (1:200, 11306-1-AP, Proteintech), p62 (1:200, ab109012, Abcam) and PINK1 (1:100, 23274-1-AP, Proteintech) were examined in each group after primary antibody incubation overnight at 4 °C and secondary antibody incubation at 37 °C for 45 min. DAPI was used to dye the nuclei. The sections were then viewed under a fluorescence microscope (Olympus). Images were captured at a magnification of 400X.

### Western blot

The ipsilateral brain tissues were homogenized on ice with lysis buffer and protein concentration were measured using BCA method. Equal amounts (40 µg) of protein extracts were subjected to 10%-12% SDS-PAGE and transferred to polyvinylidene fluoride membranes (Millipore). The membranes were incubated overnight at 4 °C with the following primary antibodies individually: anti-MnSOD (1:1000), anti-Apaf-1 (1:1000), anti-Caspase 3 (1:1000), anti-cleaved Caspase 3 (1:1000), anti-Cyto c (1:1000), anti-LC3B (1:1000), anti-Beclin1 (1:1500), anti-p62 (1:2000), and anti-PINK1 (1:1000). Then, the membranes were incubated with secondary antibodies for 1 h at room temperature. Target protein bands were captured using the BIO-RAD Imaging System with chemiluminescence detection reagents. Semi-quantitative results were obtained by measuring the optical density of the target bands and expressed as the ratio of each targeted protein to β-actin.

### Electron Microscopic Studies

The brain sections were post-fixed with 4% glutaraldehyde in 0.1 mol/L cacodylate buffer (pH 7.4). The sections were then soaked in 1% osmium tetroxide in 0.1 M cacodylate buffers for 2 h and stained with 1% aqueous uranyl acetate overnight. Tissue sections were dehydrated in ascending series of ethanol to 100% followed by acetone and embedded in epoxy resin. Ultrathin sections were counterstained with lead citrate before examination by transmission electron microscope (H7650).

### Statistics

All data are presented as means ± SD. Statistical analysis was performed using one-way ANOVA with SPSS 19.00. Student's t-test was used to analyze the difference in infarct volume between the WT and UCP2^-/-^ groups. Statistical significance was determined as *p* < 0.05.

## Results

### UCP2 deletion aggravated cerebral I/R damage

**Figure 1:**
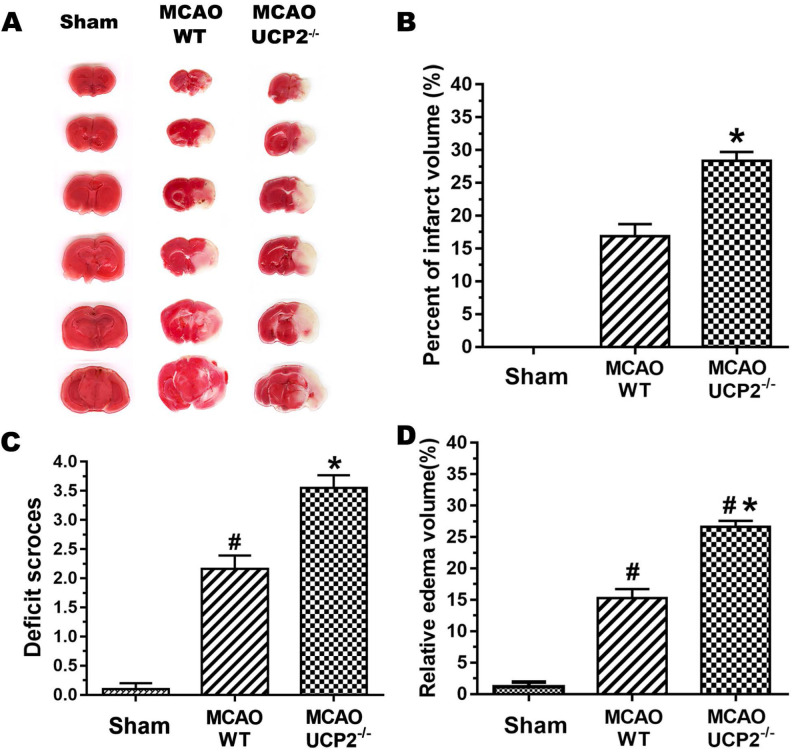
** UCP2 deletion aggravated ischemic brain damage.** (A) Representative TTC stained brain sections showing infarct volume (white color) at 24-h of reperfusion following 60min of MCAO in WT and UCP2^-/-^ mice (n=5 in each group). (B) Bar graph summarizes the mean values of cerebral infarction in WT and UCP2^-/-^ mice (n=5 in each group). Infarct volume enlarged significantly in UCP2^-/-^ mice. (C) Assessments of neurological deficits (n=24 in each group). (D) Quantitative analysis of edema volume (n=5 in each group). ^#^*p*<0.05 vs. Sham and *p<0.05 vs. WT, respectively.

UCP2 deletion enlarged infarct volume, worsened neurological deficit score and increased brain edema after cerebral I/R injury (Figure 1). The infarct volume of the mouse brain was observed by TTC staining (Figure 1A). Focal ischemia of 60 min duration induced brain infarct in the striatum and the overlaying cortex in WT mice at 24-h of reperfusion. The infarct volume was significantly enlarged in UCP2^-/-^ mice compared with the WT mice (Figure 1B). This result shows UCP2 deletion increased infarct volume. Similarly, UCP2^-/-^ mice showed a significant increase in neurological deficit score compared with WT mice (Figure 1C), indicating that neurological deficit scores were correlated to the infarct size. Measurement of brain edema also suggested that UCP2^-/-^ significantly increased brain edema compared to WT mice (*p*<0.05, Figure 1D). Combined, our data demonstrate that UCP2 deletion aggravates ischemic brain damage.

### UCP2 deletion exacerbated neural cell injury in I/R brain

**Figure 2:**
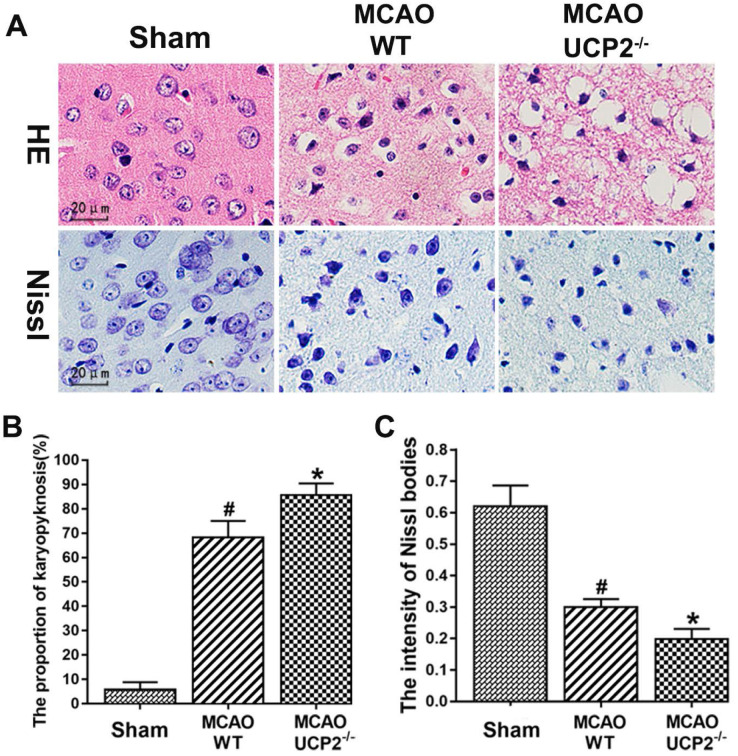
** UCP2 deletion enhanced neural cell injury after I/R in the brain.** (A) Representative images of HE staining (upper panel) and Nissl staining (lower penal). (B) Quantitative summary of pyknotic cells for HE staining (n=6 in each group). (C) The average optical density of Nissl bodies in each group (n=6 in each group). ^#^*p*<0.05 vs. Sham and *p<0.05 vs. WT, respectively.

The pathological outcomes assessed by HE and Nissl staining in the cortex after 60 min of MCAO and 24h reperfusion are given in Figure 2. As shown in the Figure 2A upper panel, a few scattered dead neurons were observed in the sham-operated animals. Transient cerebral ischemia resulted in a mildly increased number of dead neurons in the cortex after 24h of reperfusion in WT mice (*p*<0.01). As expected, deletion of UCPs further increased the percentage of dead neurons in the cortex 24h after reperfusion in comparison with the WT counterpart (Figure 2A upper panel and 2B). Similarly, Nissl staining showed I/R in WT mice reduced the density of Nissl body and deletion of UCP2 further decreased Nissl body's density compared with ischemic WT mice (Figure 2A lower panel and 2C). These data suggest that the UCP2 deletion induces neural cell damage in I/R brain.

### UCP2 deletion aggravated apoptotic death after I/R injury

**Figure 3:**
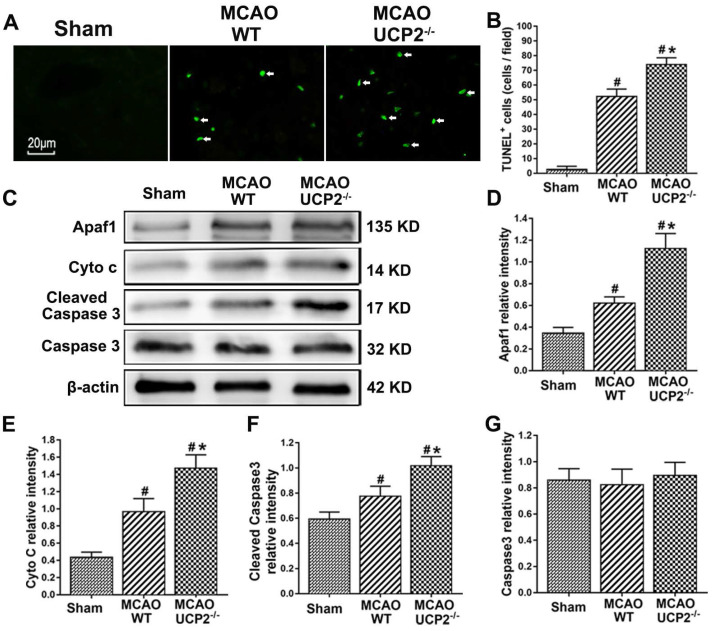
**UCP2 deletion aggravated apoptotic death after I/R injury.** (A) Representative micrographs showing TUNEL staining for apoptotic cells (green color) among different groups as indicated. Arrows indicate TUNEL-positive cells. Magnification, 400×. Scale bar = 20 µm. (B) Quantitative determination of TUNEL-positive cells in the brain cortex among different groups as indicated (n=6 in each group). Data are presented as the number of TUNEL-positive cells per high power field (HPF). (C) Representative Western blots of Apaf1, Cyto c, cleaved Caspase 3 and Caspase 3. (D-G) Semi-quantification of Apaf1, Cyto c, cleaved Caspase 3 and total Caspase 3 protein bands, respectively (n=6 in each group). Data are shown as mean ± SD. ^#^*p*<0.05 vs. Sham, *p<0.05 vs. WT, respectively.

UCP2 deletion increased the number of TUNEL positive cells and activated mitochondria-mediated cell death pathway including Apaf1, Cyto c, and cleaved Caspase 3 after I/R (Figure 3). Cellular apoptosis in ischemic mouse brain was detected with TUNEL staining (Figure 3A). TUNEL staining results revealed that I/R increased the number of TUNEL positive cells in WT mice. UCP2 deletion further elevated the numbers of TUNEL positive cells after I/R in UCP2^-/-^ compared with the WT counterparts (Figure 3A and 3B). To further examine the effect of UCP2 deletion on apoptotic pathway after I/R injury *in vivo*, we measured protein levels of Apaf1, Cyto c, cleaved Caspase 3, and total Caspase 3 in ipsilateral brain tissue collected at 24h of reperfusion using Western blotting. The results showed that I/R in WT mice caused significant increases of Apaf1, Cyto c, and cleaved Caspase 3, while total Caspase 3 was not altered. Deletion of UCP2 further elevated the levels of Apaf1, Cyto c, and cleaved Caspase 3 (Figure 3C-3G). These results suggest that deletion of UCP2 promotes brain cell apoptosis in cerebral I/R damage.

### UCP2 deletion enhanced ROS production after I/R injury

**Figure 4:**
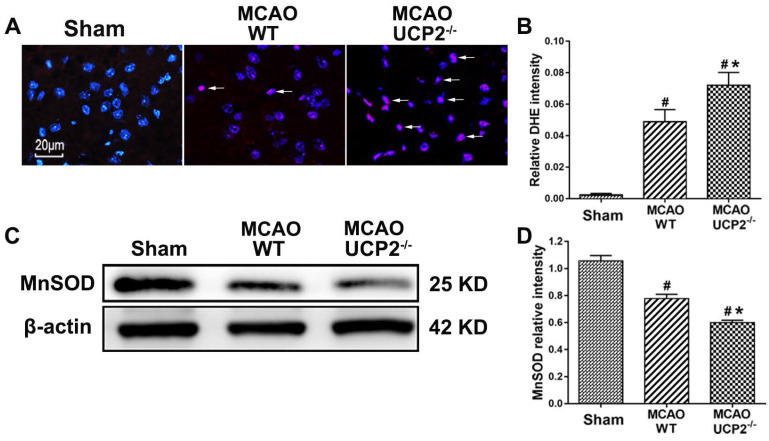
** UCP2 deletion enhanced ROS production and decreased MnSOD after focal cerebral I/R.** (A) Superoxide radicals measured by dihydroethidium (DHE) fluorescent prob in WT and UCP2^-/-^ mice (n=4 in each group). Nuclei were labeled with DAPI. Magnification, 400×. Scale bar = 20 µm. (B) Summarized DHE fluorescent intensity (n=6 in each group). Data are presented as means ± SD. ^#^*p*<0.05 vs. Sham, *p<0.05 vs. WT. (C, D) Representative Western blots and quantitative graphs demonstrate the levels of MnSOD in different groups (n=6 in each group). Data are shown as mean ± SD (n=6). ^#^*p*<0.05 vs. Sham, *p<0.05 vs. WT, respectively.

Because UCP2 has been implicated in dissipating the mitochondrial proton gradient by transporting H^+^ across the inner membrane, thereby reducing ROS production, we measured the superoxide radical levels using DHE probe in WT and UCP2^-/-^ mice to assess whether deletion of UCP2 would increase the ROS production. As shown in Figure 4A & 4B, MCAO induction caused a significant enhancement of superoxide production. Consistently, UCP2 deletion further increased the ROS compared with WT mice after I/R damage. Next, we detected the protein level of MnSOD in mouse brain by Western blot (Figure 4C). The quantitative analysis showed that the MnSOD protein level was lower in the WT group compared with the Sham group, while UCP2 deletion further decreased the level of MnSOD, when it was compared with WT group (Figure 4D). These results suggest that UCP2 deletion enhanced ROS production in the brain after I/R injury.

### UCP2 deletion activated autophagy signaling after I/R injury

**Figure 5:**
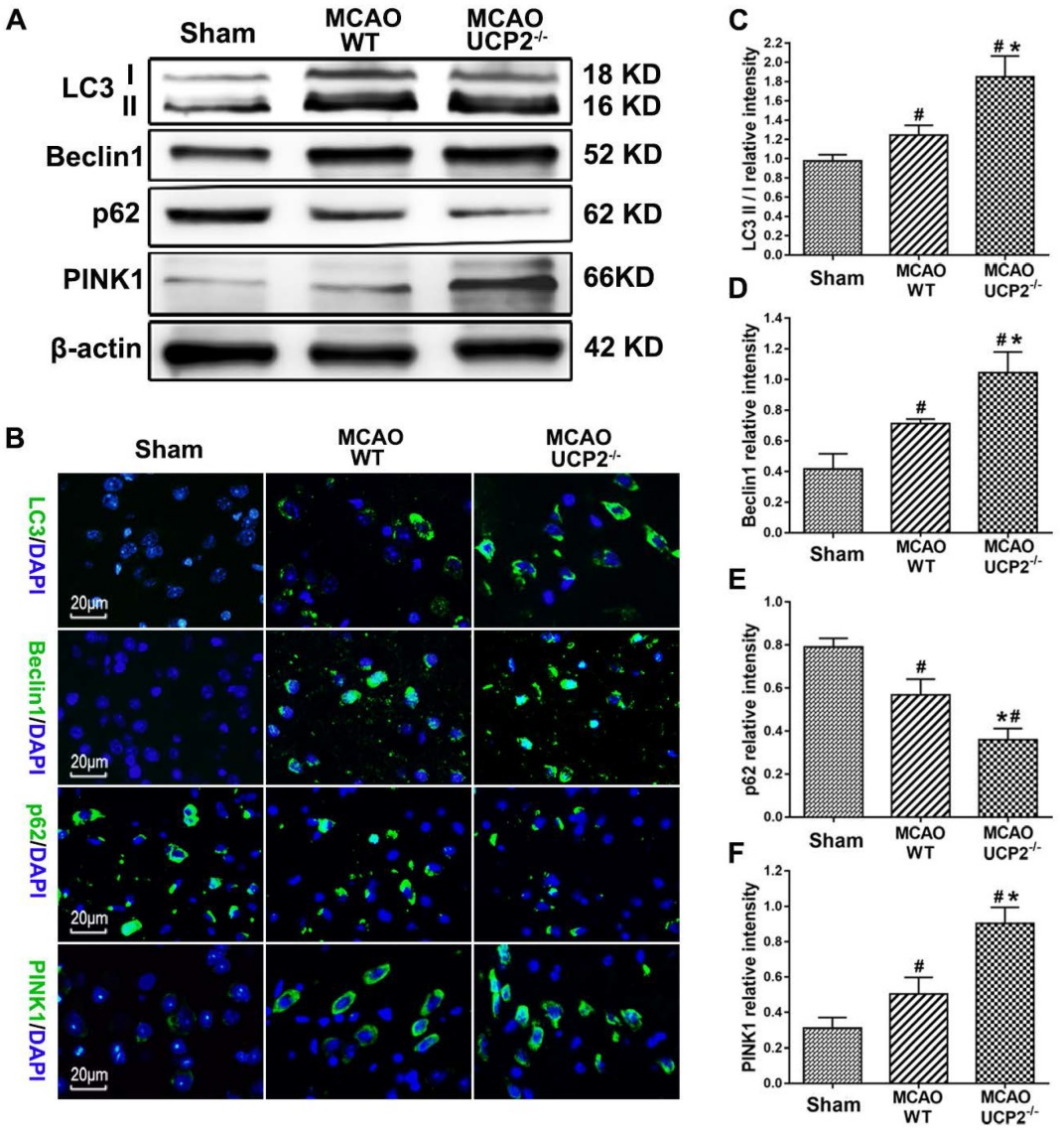
** UCP2 deletion activated autophagy pathway after I/R injury.** (A) Representative Western blots of LC3-B, Beclin-1, p62, and PINK1 in brain samples collected from peri-infarct area at 24 h after I/R. (B) Immunohistochemistry of LC3-B, Beclin-1, p62, and PINK1 (green color). Sections were counter labeled with DAPI (Blue color) to mark nuclei. Scale bar = 20 µm. (C-F) Bar graphs demonstrate semi-quantitations of LC3-II/LC3-I, Beclin-1, p62, and PINK1 (n=6 in each group). Data are shown as mean ± SD. ^#^*p*<0.05 vs. Sham and *p<0.05 vs. WT.

Autophagy is one of a major cell death patterns. To investigate whether deletion of UCP2 would activate autophagy signaling pathway after cerebral I/R injury, we measured the levels of major autophagy regulators including LC3-B, Beclin-1, p62, and PINK1 using Western blots and immunohistochemistry in brain samples collected from the peri-infarct area after 24 h of reperfusion following 60 min of MCAO (Figure 5A). The results showed I/R increased the protein levels of LC3 II, Beclin-1 and PINK1, while it decreased the level of p62, in WT animals (*p<*0.05 vs. Sham). Compared with WT, I/R in UCP2^-/-^ mice further increased LC3 II, Beclin-1 and PINK1 and decreased p62 (Figure 5A, 5C-5F). Immunolabeling confirmed the trend changes of LC3, Beclin-1, PINK1 and p62 (Figure 5B). These results suggest that cerebral I/R activates autophagy pathway and deletion of UCP2 augments the I/R-induced activation.

### UCP2 deletion induced autophagosome accumulation in cortex neurons after I/R injury

**Figure 6:**
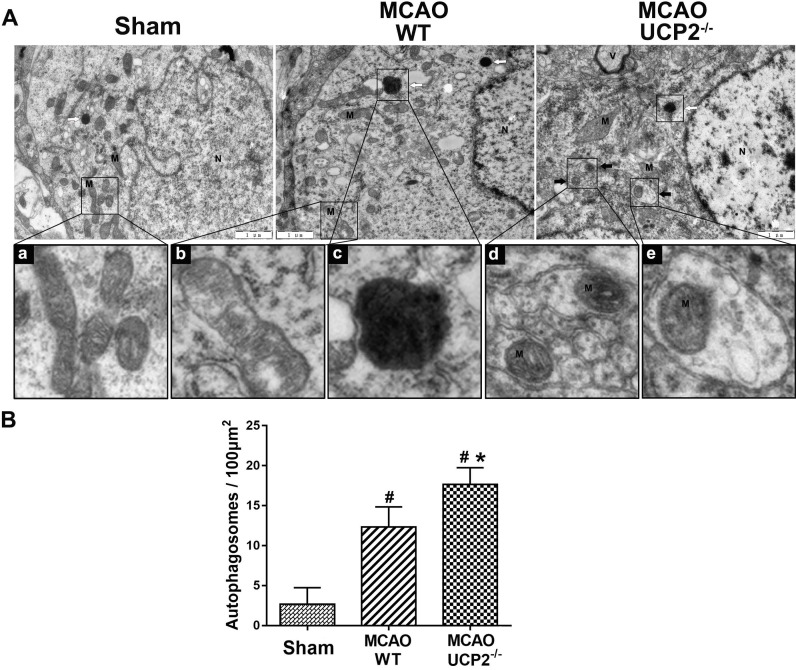
** The transmission electron microscope (TEM) examination of ultrastructural changes in cortical neurons 24h after the reperfusion in WT and UCP2^-/-^ mice.** (A) Representative microphotographs showing autophagosomes in different groups (upper panel). The high magnification images from the cropped rectangle are given in lower panel, in which (a) Normal mitochondrion, (b) mild mitochondrial swollen, (c) autolysosomes, and (d, e) typical mitophagy structure. The characteristics of mitophagy structure (black arrow) and autolysosome structures (white arrow) were observed in neurons 24 h after MCAO in UCP2^-/-^ mice. M, mitochondria; N, nuclei; V, vacuole. scale bar = 1 µm. (B) Quantification of autophagosomes per 100 μm^2^ in neuron (n=3 in each group). Data are shown as mean ± SD. ^#^*p*<0.05 vs. Sham, *p<0.05 vs. WT.

It was not clear whether activation of autophagy signaling pathway would actually induce autophagy in the brain after I/R injury. To demarcate the effect of UCP2 deletion on autophagy after cerebral I/R, we performed transmission electron microscopy to examine autophagosomes and autolysosome structures in neurons. As shown in Figure 6, neuron in Sham-operated normal animals showed normal appearance of nucleus (N), abundant endoplasmic reticulum and relatively healthy looking mitochondria (M). In contrast, neuron in the peri-ischemic cortex of the WT ischemic animals displayed mild swollen mitochondria (b) and abnormal autolysosomes (c) at 24 h after I/R. Some autophagosomes were also observed in WT group. Furthermore, in the neurons of the UCP2^-/-^ animals, we observed many double layered membrane structures with enclosed mitochondria, which is typically defined as mitochondrial autophagy or mitophagy. Quantitation of autophagosomes in each animal group revealed that I/R in WT significantly increased the number of autophagosome and UCP2 deletion further increased the number compared with WT ischemic animals (Figure 6B). These results indicate that UCP2 deletion is associated with induction of mitophagy in the brain following I/R injury.

## Discussion

The present study demonstrated that deficiency of UCP2 significantly increased infarct volume and brain edema and caused more severe neurological deficit scores after I/R in UCP2^-/-^ mice than WT mice subjected to identical length of I/R. Further, UCP2 deletion enhanced ROS production and cell apoptosis. Moreover, deletion of UCP2 elevated LC3-II/LC3-I ratio, Beclin1 and PINK1 protein contents, suppressed p62 protein level, and increased the number of mitochondria containing autophagosomes compared with respective WT ischemic controls. These results suggest that UCP2 deletion may exacerbate cerebral I/R injury via reinforcing mitophagy and cell apoptosis.

UCP2 is a member of inner mitochondrial membrane proteins, which stabilizes the inner mitochondrial membrane potential and reduces ROS generation 1, 2. UCP2 is widely expressed in various tissues including central nervous system. Emerging evidence suggests that UCP2 plays multiple roles in cerebral I/R injury. On the one hand, it has been found that UCP2 regulates mitochondrial potential, energy balance, and ROS generation; on the other hand, UCP2 has been shown to be associated with cell death and inflammation 18. It has been reported that overexpressing UCP2 has neuroprotective effects in cerebral stroke 19. Our data demonstrated that the genetic ablation of UCP2 significantly increased infarct volume, brain edema, and aggravated neurological deficit scores after I/R, which is consistent to our previous report and those published in the literature 6, 20, 21. The previous study has demonstrated that UCP2 induces mitochondrial changes, i.e., increase in mitochondrial density and reduction in mitochondrial size, suggesting that UCP2 expression is associated with a mitochondrial fission process in neurons 22. And our lab also found that UCP2 ablation disturbed mitochondrial dynamic balance tilting it towards fission in cerebral I/R damage 23. Furthermore, our results demonstrated that UCP2 deletion further increased neural cell death in I/R brain as assessed by histological studies. These results suggest that UCP2 plays an important role in cerebral I/R injury.

Mitochondrial reactive oxygen species (ROS) and apoptosis have been demonstrated to play a critical role in cerebral ischemic/reperfusion injury. ROS are free radicals that can damage DNA, lipids, membranes and organelles, which can lead to activation of cell death processes such as apoptosis 24. There is abundant evidence showing that the ROS are the by-products of cellular metabolism or of xenobiotic exposure. ROS not only play important roles in cellular signaling but also cause cell damage 25. It has been reported that UCP2 reduces the ROS formation by preventing mitochondrial membrane hyperpolarization that in turn inhibits mitochondrial electron transport chain. Studies have shown that UCP2 confers protective effects on various stressors by decreasing mitochondrial ROS production in the brain and cardiomyocytes 26-29. MnSOD is encoded by nuclear genome, synthesized in the cytosol, transported into the mitochondrial matrix post translationally via TOM and TIM23 complexes, and assembled into active enzyme with the incorporation of a manganese ion in the mitochondrial matrix. The mitochondrial import machinery is a susceptible cellular target of superoxide radicals 30. The decreased MnSOD enzyme observed in UCP2^-/-^ I/R animals is probably due to increased ROS in UCP2^-/-^ mouse damaged the mitochondrial importing carriers and decreased the transportation of MnSOD from the cytosol into the mitochondrial. Another possibility is that enhance ROS production consumed large amount of MnSOD. In the present study, UCP2 deletion partially induced mitochondrial dysfunction and suppressed MnSOD expression, then enhanced ROS accumulation in the brain after I/R.

Apoptosis is one of the pathways of cell death that are activated by cerebral I/R injury. There is abundant evidence showing that the mitochondria-initiated apoptotic pathway is activated in response to a variety of cellular stresses, including ROS 31, 32. Indeed, mitochondria are the major site where most intracellular ROS are produced and accumulation of ROS can trigger the formation of mitochondria permeability transition pore (MPTP). The MPTP allow releases of pro-apoptotic factors such as Apaf-1 and cytochrome c (Cyto c) from the mitochondria to the cytosol, where activating caspases and eventually causing apoptotic cell death 33, 34. In this study, we found that cerebral I/R increased the protein levels of apoptosis-associated proteins that include Apaf1, Cyto c and cleaved Caspase 3 after I/R injury, and UCP2 deletion further augmented the elevations of these proteins. These results show that deletion of UCP2 further exacerbates the activation of mitochondria-mediated cell death pathway and the resulting apoptotic cell death in the brain after cerebral ischemic injury. In the meantime, we also found that the presence of high levels of ROS after I/R damage in UCP2^-/-^ mice. These results support the hypothesis that UCP2 deletion activates apoptotic cell death probably through increasing ROS after cerebral I/R. Our result is supported by previously published data showing that over-expression of UCP2 decreases ROS and apoptosis in cardiomyocytes and in the brain 7, 8, 35.

Mitochondrial autophagy (mitophagy) is one of the most important types of autophagy in the central nervous system. It refers to selective sequestration of mitochondria by autophagosomes and subsequently delivers them to lysosomes for destruction and material recycle 10. Previous studies have shown that mitophagy is mainly evident in neurons 36 and astrocytes 37 of the ischemic brain. However, it is controversial whether I/R-triggered mitophagy is beneficial or detrimental to the tissue. On the one hand, inhibition of mitophagy has been shown to exacerbate or reduce ischemic brain injury; on the other hand, enhancement of mitophagy led to cell death 13, 14, 15. In other words, insufficient or excessive mitophagy may cause cell death due to damaged mitochondrial accumulation or removal of indispensable mitochondria. LC3, Beclin-1, p62, and PINK1 are key regulators for induction of autophagy and mitophagy. LC3 is the most widely used marker for revealing the presence of autophagosomes during autophagy and mitophagy activation. Autophagosome formation involves the conversion of LC3 from LC3-I to LC3-II, suggesting that the LC3-II/LC3-I ratio closely correlates with the number of autophagosomes 38. Beclin-1 is another important molecule for mitophagy, which involved in the formation of autophagosomes by membrane recruitment 39. Additionally, mutations in the mitochondrial Ser/Thr kinase PTEN-induced kinase 1 (PINK1) participates PINK1/Parkin-mediated mitophagy, which is the best-characterized mechanism for elimination of damaged mitochondria by mitophagy in mammalian cells 40, 41. In damaged mitochondria, PINK1 is stabilized on the outer mitochondrial membrane and recruits Parkin and activates latent E3 ligase activity of Parkin 42, 43. Parkin activation leads to proteasomal degradation of outer mitochondrial membrane proteins and to mitophagy 44, 45. Recent studies have shown that mitophagy interacts with ROS and apoptosis in stroke, especially in PINK1/Parkin-mediated mitophagy 13, 15. In the present study, we used western blots and immunohistochemistry staining to detect LC3, Beclin-1, and PINK1 expression in the ischemic/reperfusion brain. We found that the LC3-II/LC3-I ratio, Beclin1 and PINK1 protein level were raised after cerebral ischemic/reperfusion. Deletion of UCP2 further increased the levels of LC3-II/LC3-I conversion, Beclin1 and PINK1 compared with WT ischemic control. The p62 (aka SQSTM1), a vital cargo receptor in pink1/parkin mediated mitophagy pathway, is mainly located in the cytosol and forms aggregates 46. When mitophagy is activated, the p62 protein translocates to the mitochondria and localizes on the sites of autophagosome. It binds with autophagosome localizing protein LC3 and/or ubiquitinated proteins to degrade the autophagy target such as damaged mitochondria 46, 47. In this study, we detected the expression of p62 by western blot assays. The results showed that the p62 protein level decreased after cerebral I/R and UCP2 deletion further reduced it. The evidence presented in this study indicates that UCP2 deletion reduces accumulation of p62, which is consistent with previous study 11. The increases of autophagy markers together with mitophagy associated protein marker suggest UCP2 deletion increases mitophagy. The follow up electron microscopic study confirmed the existence of mitophagy in UCP2^-/-^ animals subjected to I/R.

To study the mitochondrial morphology and to confirm whether activation of mitophagy pathway would lead to increased mitophagy, we performed electron microscopic studies. The results demonstrated that the mitochondrial ultrastructural alterations were much prominent in UCP2^-/-^ mice. Further, abnormal mitochondria fused with autophagic vesicles or surrounded by double membranes of typical autophagosomes were observed in UCP2^-/-^ animals. Quantitative analysis of autophagosomes showed that the number of mitochondria-containing autophagosomes in UCP2^-/-^ group was higher than that in the WT group. Considering the increased mitophagy in UCP2^-/-^ mice is accompanied with increased ROS, activated cell death pathway, and augmented neuronal death, we believe that increased mitophagy observed in UCP2^-/-^ animals after I/R is detrimental rather than beneficial. This view is supported by Yang and colleagues who report cerebral ischemic damage is associated with excessive autophagy 48.

In conclusion, the present study demonstrated that deletion of UCP2 gene further exacerbated I/R-induced brain damage, apoptotic cell death, ROS production and mitophagy. The increased mitophagy is associated with worse ischemic outcome, suggesting excessive mitophagy is harmful in I/R setting.

## Highlights

- Deletion of mitochondrial uncoupling protein 2 (UCP2) exacerbates brain damage after cerebral ischemia/reperfusion injury in mice;
- Deletion of UCP2 aggravates apoptotic death and ROS production after ischemia/reperfusion injury;
- Deletion of UCP2 enhances mitochondrial autophagy (mitophagy) signaling after ischemia/reperfusion injury.

## Abbreviations

UCPs: Uncoupling proteins
UCP2: Uncoupling protein 2
UCP2^-/-^: UCP2 deletion
I/R: ischemia/reperfusion
MCAO: middle cerebral artery occlusion
WT: wildtype
ROS: reactive oxygen species
TUNEL: Terminal deoxynucleotidyl transferase mediated dUTP nick‑end labeling
TTC: 2,3,5-Triphenyl Tetrazolium Chloride
ICA: internal carotid artery
ECA: external carotid artery
CCA: common carotid artery
DHE: Dihydroethidium
